# Mismatch of thermal optima between performance measures, life stages and species of spiny lobster

**DOI:** 10.1038/s41598-020-78052-4

**Published:** 2020-12-04

**Authors:** Samantha Twiname, Quinn P. Fitzgibbon, Alistair J. Hobday, Chris G. Carter, Michael Oellermann, Gretta T. Pecl

**Affiliations:** 1 Institute for Marine and Antarctic Studies, University of Tasmania, Hobart, TAS Australia; 2 CSIRO Oceans and Atmosphere, Hobart, TAS Australia; 3 Aquatic Systems Biology Unit, Technical University of Munich, Freising, Germany

**Keywords:** Ecology, Physiology, Zoology

## Abstract

In an ocean warming hotspot off south-east Australia, many species have expanded their ranges polewards, including the eastern rock lobster, *Sagmariasus verreauxi*. This species is likely extending its range via larval advection into Tasmanian coastal waters, which are occupied by the more commercially important southern rock lobster, *Jasus edwardsii*. Here, thermal tolerances of these lobster species at two life stages were investigated to assess how they may respond to warming ocean temperatures. We found that the pattern, optimum and magnitude of thermal responses differed between performance measures, life stages and species. *Sagmariasus verreauxi* had a warmer optimal temperature for aerobic scope and escape speed than *J. edwardsii*. However, *J. edwardsii* had a higher magnitude of escape speed, indicating higher capacity for escape performance. There were also differences between life stages within species, with the larval stage having higher variation in optimal temperatures between measures than juveniles. This inconsistency in performance optima and magnitude indicates that single performance measures at single life stages are unlikely to accurately predict whole animal performance in terms of life-time survival and fitness. However, combined results of this study suggest that with continued ocean warming, *S. verreauxi* is likely to continue to extend its distribution polewards and increase in abundance in Tasmania.

## Introduction

Ocean warming is causing widespread alterations to species’ geographical ranges in marine communities worldwide^1–6^. However, the pace at which species alter their distribution occurs at different velocities, likely changing their interaction with other species, with ramifications for food webs and potentially widespread modifications of ecosystems^7–10^. These changes in distribution have implications for socioeconomic productivity and human health^1,11^. Understanding if and how species may shift their range under warming scenarios and how species interactions may change, is a critical step to understanding the complex effects of climate change on marine ecosystems and improving predictive capacity.

Thermal performance curves have been a common approach to assess species responses to temperature and more recently to climate warming^12–14^. Thermal performance curves map changes of a particular performance measure (e.g. metabolism, growth) against changes in temperature to derive a species’ optimal or suboptimal temperatures for these specific measures^14–17^. This approach is particularly useful for ectothermic organisms whose body temperature and associated body functions are directly affected by ambient temperature^12,15^. These measures of performance can be any temperature dependent biological rate such as metabolism, growth, reproduction and escape speed^18–20^. Ecologically relevant thermal performance curves could inform and help predict potential changes in organism performance in a changing climate and facilitate predictions of future range shifts.

While thermal performance curves are used to predict thermal sensitivity in a single species, they may also be a useful tool to predict the potential changes in outcomes of species interactions. Species range shifts can alter species interactions, such as predator prey relationships or competitive interactions for resources, in different ways^21–23^. These include the development of novel species interactions due to asynchronous distribution shifts, where some organisms extend their ranges to new areas, that could create new interactions with the resident species^8,24^. Another is the modification of existing interactions, through changes in the abundance of species, or changes in relative performance of one or both species^25^. It is unlikely that both species in an interaction will react to changes in temperature the same way and hence asymmetries will arise in the response and outcomes of interactions^22,26^. Depending on the geographic location within a species range and its individual thermal boundaries, physiological and ecological performance may increase or decrease. For example, at the warm edge of a species range, further warming may reduce performance of measures such as swimming performance^27^. In contrast, at the cool end of their range, warming may increase swimming performance^28,29^. Using thermal performance curves will help identify how individual species react to changes in ocean temperatures and potentially inform at what temperature the outcomes of species interactions may alter.

One common measure of physiological performance used to investigate effects of temperature is aerobic scope^16,30,31^. Aerobic scope is the difference between maximum- and standard oxygen consumption rates^30^ and estimates the amount of aerobic energy available—in excess of that consumed by basic body functions—to support other important activities. The use of aerobic scope measurements to predict whole organism performance changes under ocean warming has been widely implemented and discussed, with support for and against its use^16,31–33^. The oxygen and capacity-limited thermal tolerance hypothesis proposes aerobic scope as a unifying proxy for animal fitness and suggests that optimal performance aligns with temperatures at which aerobic scope becomes maximal^16^. However, there is increasing evidence from recent studies indicating that aerobic scope may not be sufficient to predict optimal animal performance^19,20,34^. Despite aerobic scope not necessarily being an overarching mechanism as proposed by the oxygen and capacity-limited thermal tolerance hypothesis^31^, it is still a useful metabolic performance measure that can provide insight into the energy available for aerobic activities^35^. Combined with other performance measures, it can provide information on species performance or fitness along a temperature gradient.

Escape speed presents another ecologically relevant performance measure that changes with temperature and is acutely critical to survival, often within a matter of seconds^28,36,37^. If a species escape speed changes with ocean warming, outcomes between predator and prey may change, which may cause major shifts in the structure and function of an ecosystem^22,26,38^. In contrast to aerobic scope, escape bursts are mostly powered anaerobically by white muscle and linked to aerobic metabolism only during recovery^39–41^. For most species it is unknown whether optimal temperatures for two critical but independent performance measures—aerobic scope and escape speed—align, co-vary or mismatch. Determining the outcome of this question may support a more robust assessment of species current and future range shifts using performance measures most appropriate to the life stage, or species.

**Figure 1:**
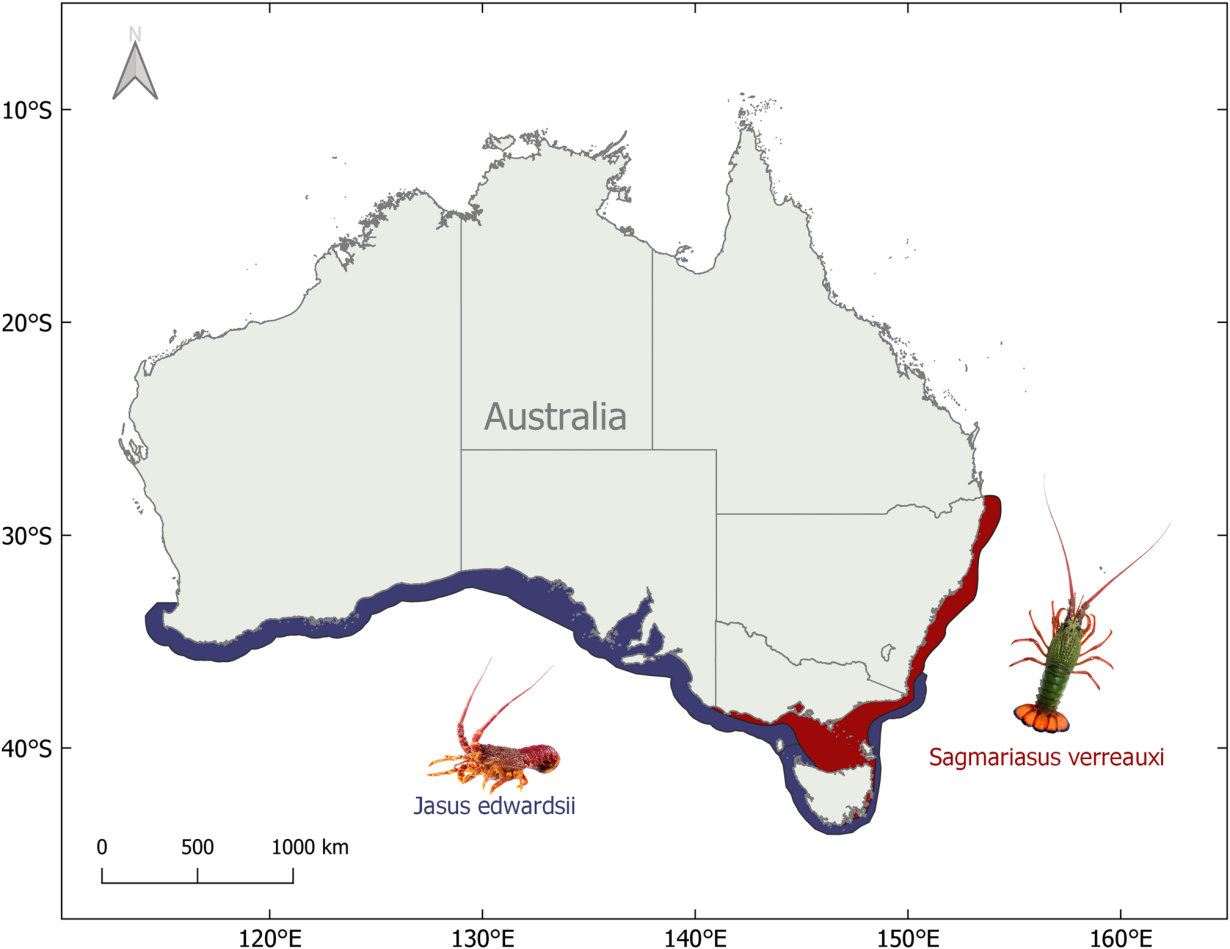
Map of the historical geographical ranges of *Jasus edwardsii* (blue) and *Sagmariasus verreauxi* (red) in Australia. Image courtesy of Craig Mostyn Group and Seafood New Zealand.

Identified as an ocean warming hotspot, south-east Australia is warming at four times the rate of the global average^42^. This is in part due to the increase in the strength and extent of the East Australian Current, which is pushing warmer water further south along the coast^43^. As a result, species’ are shifting into areas they have never or rarely been recorded^2,3,9,44^. One of these species is the eastern rock lobster (ERL), *Sagmariasus verreauxi*, commonly found along the south-east coast of mainland Australia and sporadically in northern Tasmania. This species is considered likely to be undergoing a range-shift further into Tasmanian waters^44^. Pueruli of *S. verreauxi* have been caught sporadically in larval collection traps, and fishery legal sized lobsters (carapace length minimum 105–110 mm) are occasionally caught by both commercial and recreational fishers in Tasmania. Divers have also observed *S. verreauxi* in dens along the east coast of the state (Redmap sightings 1238, 1305, and 3534^45^). This expansion is likely facilitated by the increase in the strength and extent of the East Australian Current in south east Australia, carrying sub-tropical larvae further poleward as well as warming temperatures allowing species transported to these areas to survive winter conditions^43,46–49^. As a result, this range shift is bringing *S. verreauxi* into areas dominated by the local species, southern rock lobster (SRL), *Jasus edwardsii*. Novel interactions between the species, either through new interactions or changes in abundance of the interacting species, are possible. Both species in their home ranges occupy similar ecological niches, making competition between the species likely in areas their populations overlap. Currently, this overlap is a relatively small proportion of both of their historical geographic ranges (Fig. 1), with low abundances of *S. verreauxi* found in Tasmania. It is uncertain how increasing temperatures and a novel biotic interaction between range-shifting *S. verreauxi* and the more commercially valuable *J. edwardsii* will change their important ecological roles and respective economic benefits. Understanding their sensitivity to ocean warming and how this will affect outcomes of their mutual interaction is critical to apply sustainable management practices for both species.

To address the potential (mis)match in optima between thermal performance measures, we compared a widely applied physiological performance indicator, aerobic scope, with another ecologically important performance indicator, escape speed, at the final larval (puerulus) and juvenile life stage of *J. edwardsii* and *S. verreauxi*. It has been shown that different life stages may have different thermal tolerances^50^, with larval stages in crustacean species indicating higher sensitivity to changes as a result of warming temperatures^51–53^. Here, multiple life stages were investigated to identify any potential differing responses in performance measures. Respirometry was used to determine metabolic rates (standard, routine, and active metabolic rates) and calculate aerobic scope of individual lobsters at a range of temperatures. Recovery from exercise, including time taken to recover to a routine metabolic rate and the excess post-exercise oxygen consumption (EPOC) were also calculated. Videography was used to film the escape responses of individuals, where measures of maximum and average velocity and number of escape responses were recorded. We tested the hypotheses that (1) due to their differing climatic origins, *S. verreauxi* will have warmer thermal optima for the measured performance traits than *J. edwardsii*, (2) species’ have different thermal optima for two distinct performance measures and (3) there will be temperatures where the thermal performance curves of both species overlap, highlighting potential spatial gradients in interaction outcomes.

## Results

**Figure 2:**
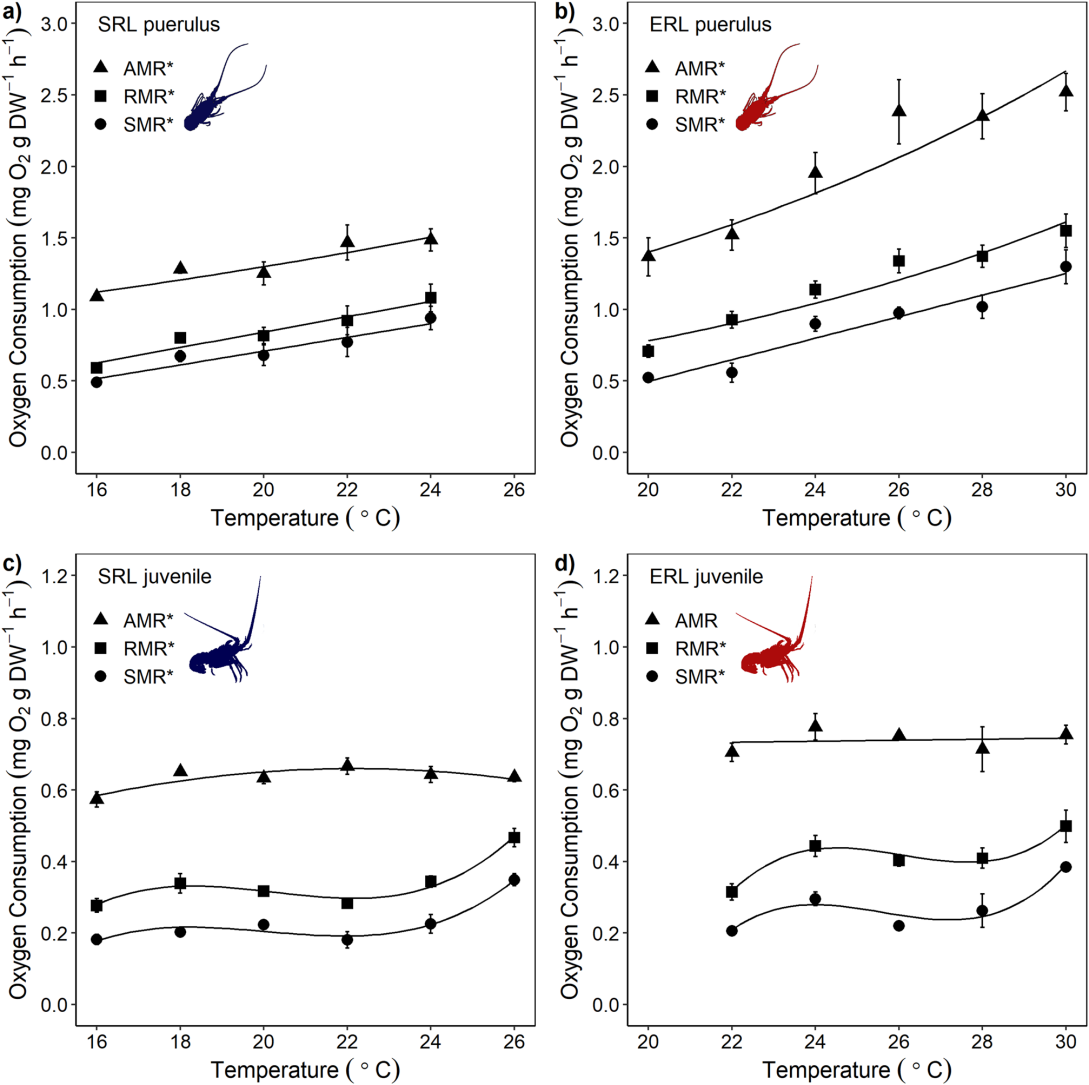
Metabolic rates of *Jasus edwardsii* (SRL) and *Sagmariasus verreauxi* (ERL) at different life stages, where (**a**) shows *J. edwardsii* pueruli, (**b**) *S. verreauxi* pueruli, (**c**) *J. edwardsii* juveniles and (**d**) *S. verreauxi* juveniles. Metabolic rates in the legends are active metabolic rate (AMR), routine metabolic rate (RMR) and standard metabolic rate (SMR). Values are mean ± 1 SE. Sample size ranged from 3 to 10 individuals per temperature treatment. Significance at α = 0.05 signified by * in legends. Details for regressions are provided in Supplementary Table S1.

Metabolic rates and escape responses were investigated using linear, generalised linear, and linear mixed effects models. As the generalised linear and linear mixed effects models did not improve the model fits as determined by AIC values, all regression models referred to herein are linear models. All metabolic rates, except the active metabolic rate for *S. verreauxi* juveniles, increased significantly with temperature across their tested thermal range (Fig. 2, Supplementary Table S1). Model relationships varied between species and life stage from linear to polynomial regressions (Fig. 2, Supplementary Table S1).

**Figure 3:**
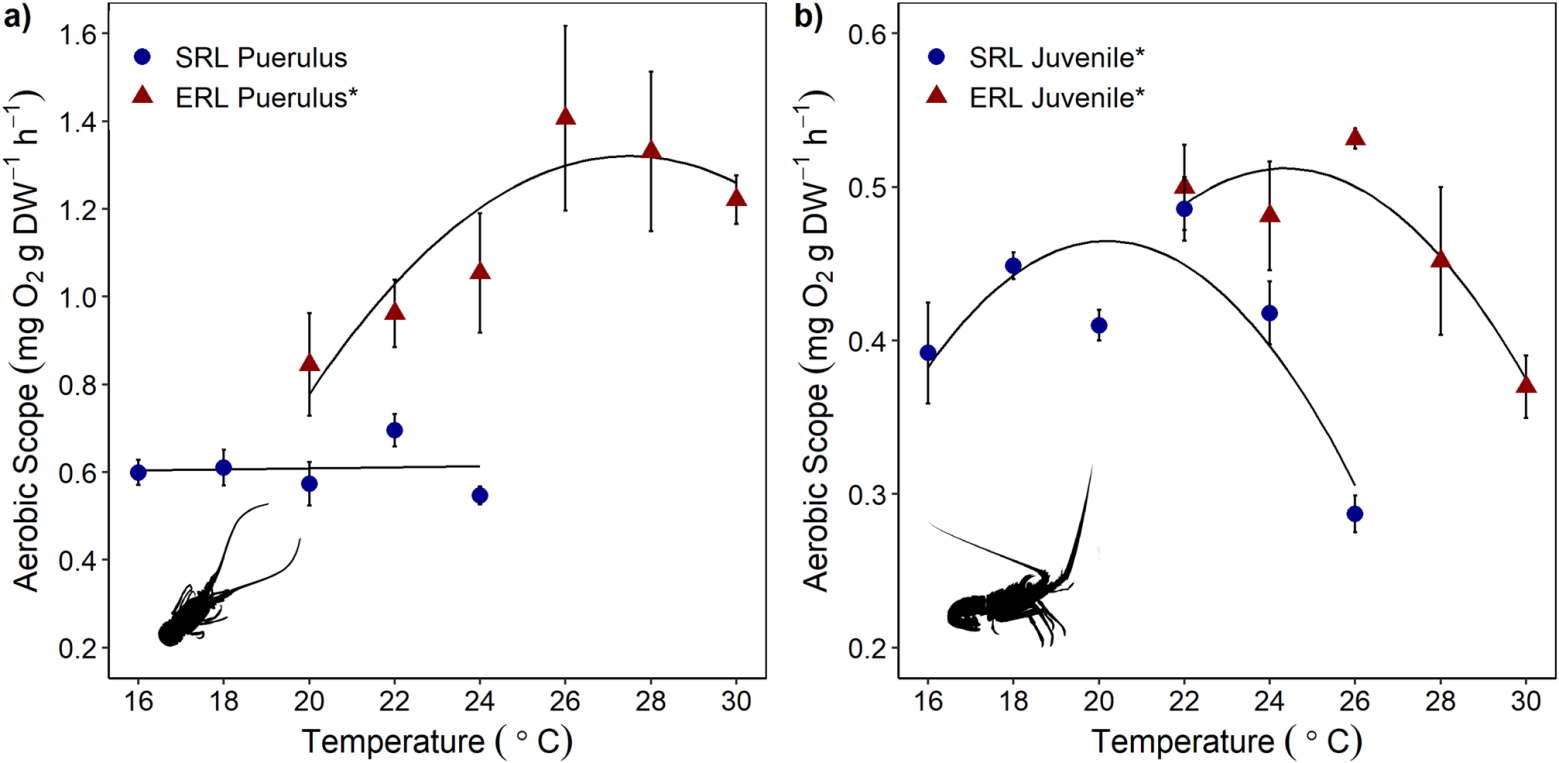
Aerobic scope of *J. edwardsii* (SRL) and *S. verreauxi* (ERL), where (**a**) indicates puerulus and (**b**) juvenile life stages. Values are mean ± 1 SE. Sample size ranged from 3 to 10 individuals per temperature treatment. Significance at α = 0.05 signified by * in legends. Details for regressions are provided in Supplementary Table S1.

Nearly all aerobic scope measurements for all species and life stages followed quadratic relationships with temperature, and all showed significant changes in response to temperature. The exception was *J. edwardsii* pueruli aerobic scope which exhibited a non-significant linear relationship with temperature (Fig. 3, *J. edwardsii* pueruli; *p* = 0.871, Supplementary Table S1). While aerobic scope did not change with temperature up to 24 °C, all three pueruli tested at 26 °C died. Two of these mortalities occurred before the chase period, and one shortly after.

**Figure 4:**
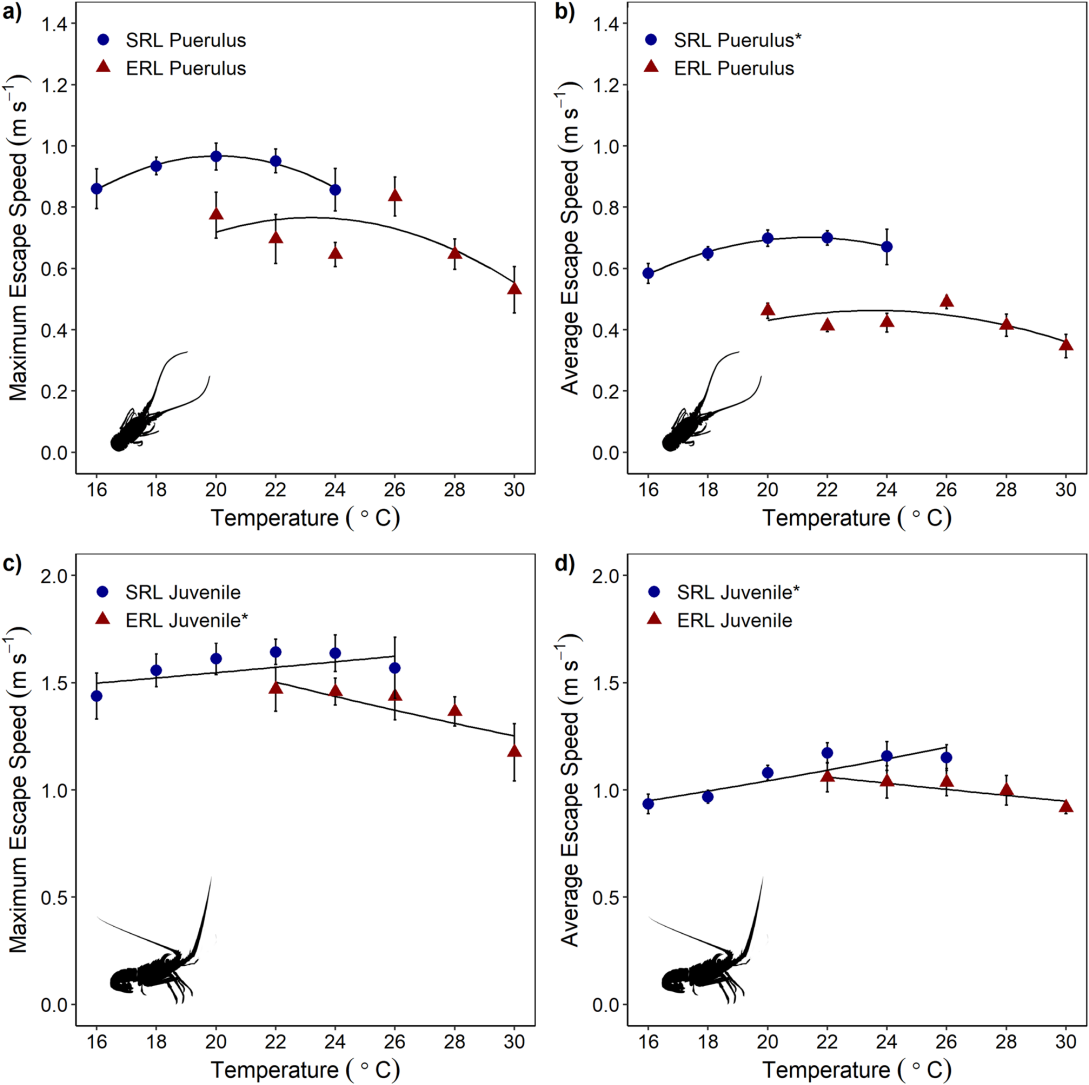
Maximum and average escape speeds of *Jasus edwardsii* (SRL) and *Sagmariasus verreauxi* (ERL), where (**a**) shows the maximum escape speed of the pueruli, (**b**) the average escape speed of the pueruli, (**c**) the maximum escape speed of the juveniles and (**d**) the average escape speed of the juveniles. Values are mean ± 1 SE. Sample size ranged from 3 to 10 individuals per temperature treatment. Significance at α = 0.05 signified by * in legends. Details for regressions are provided in Supplementary Table S1.

Escape speeds, both maximum and average speeds, displayed quadratic model regressions over the tested temperature ranges for both species and life stages (Fig. 4, Supplementary Table S1). However only the *J. edwardsii* average escape speed relationships changed significantly with temperature (regression models; pueruli *p* = 0.009, juveniles *p* < 0.001, Supplementary Table S1), though *S. verreauxi* pueruli relationships were near significant for both maximum and average escape speeds (regression models; *p* = 0.053 and *p* = 0.053, respectively, Supplementary Table S1).

**Figure 5:**
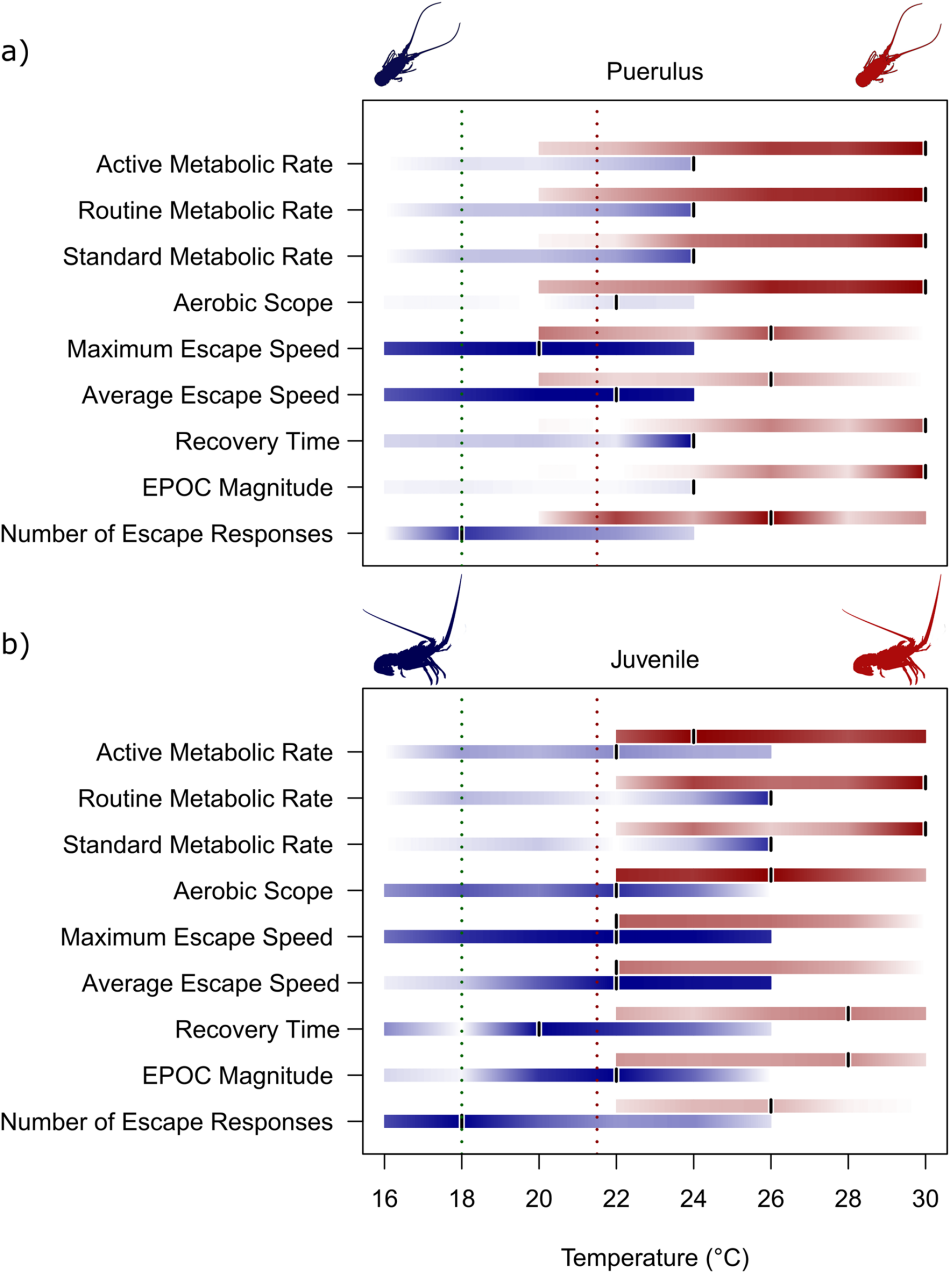
Comparison of thermal optima over a range of performance measures for *Jasus edwardsii* and *Sagmariasus verreauxi*, where (**a**) shows the pueruli measures and (**b**) the juveniles measures. In both plots, the blue bars signify *J. edwardsii* (between 16–26 °C) and the red bars *S. verreauxi* (between 20–30 °C). The intensity of the bar indicates the magnitude of performance, scaled across values for both species, where high intensity (darker coloured) represents higher performance. The black vertical bars indicate the temperature at which the highest measure of performance was achieved. The dotted lines indicate current (~ 18 °C) and future (~ 21.5 °C) temperatures in Tasmania, for context.

Thermal optima differed between species, life stage and measure of performance (Table 1, Fig. 5). Due to *J. edwardsii* puerulus aerobic scope not exhibiting a quadratic relationship with temperature required to calculate an optimal temperature, a pseudo-optimal temperature of 22 °C has been selected based on consistently higher performance compared to the other temperature treatments. While the juvenile stages of each species showed similar thermal optima between the two measures of performance, the *S. verreauxi* puerulus stage had differences in the thermal optimums between aerobic scope and maximum escape speed of ~ 4 °C. For *J. edwardsii*, thermal optima were warmer for the puerulus stage than the juvenile but were similar for each measure within life stage. For *S. verreauxi*, escape speed thermal optima were similar between life stages, but aerobic scope optima was warmer for the puerulus stage than the juvenile stage, an opposite trend compared to *J. edwardsii*.

**Table 1:** Thermal optima derived from derivative of quadratic regression models for two measures of performance for *Sagmariasus verreauxi* and *Jasus edwardsii* juveniles.

Species and life stage	Aerobic scope	Maximum escape speed
*Jasus edwardsii* puerulus	~ 22 °C	22.25 °C
*Jasus edwardsii* juvenile	19.63 °C	19.85 °C
*Sagmariasus verreauxi* puerulus	27.49 °C	23.21 °C
*Sagmariasus verreauxi* juvenile	24.34 °C	23.63 °C

Note that the underlined temperature for *J. edwardsii* puerulus aerobic scope is a pseudo optimal estimate, not calculated from a quadratic regression model.

**Figure 6:**
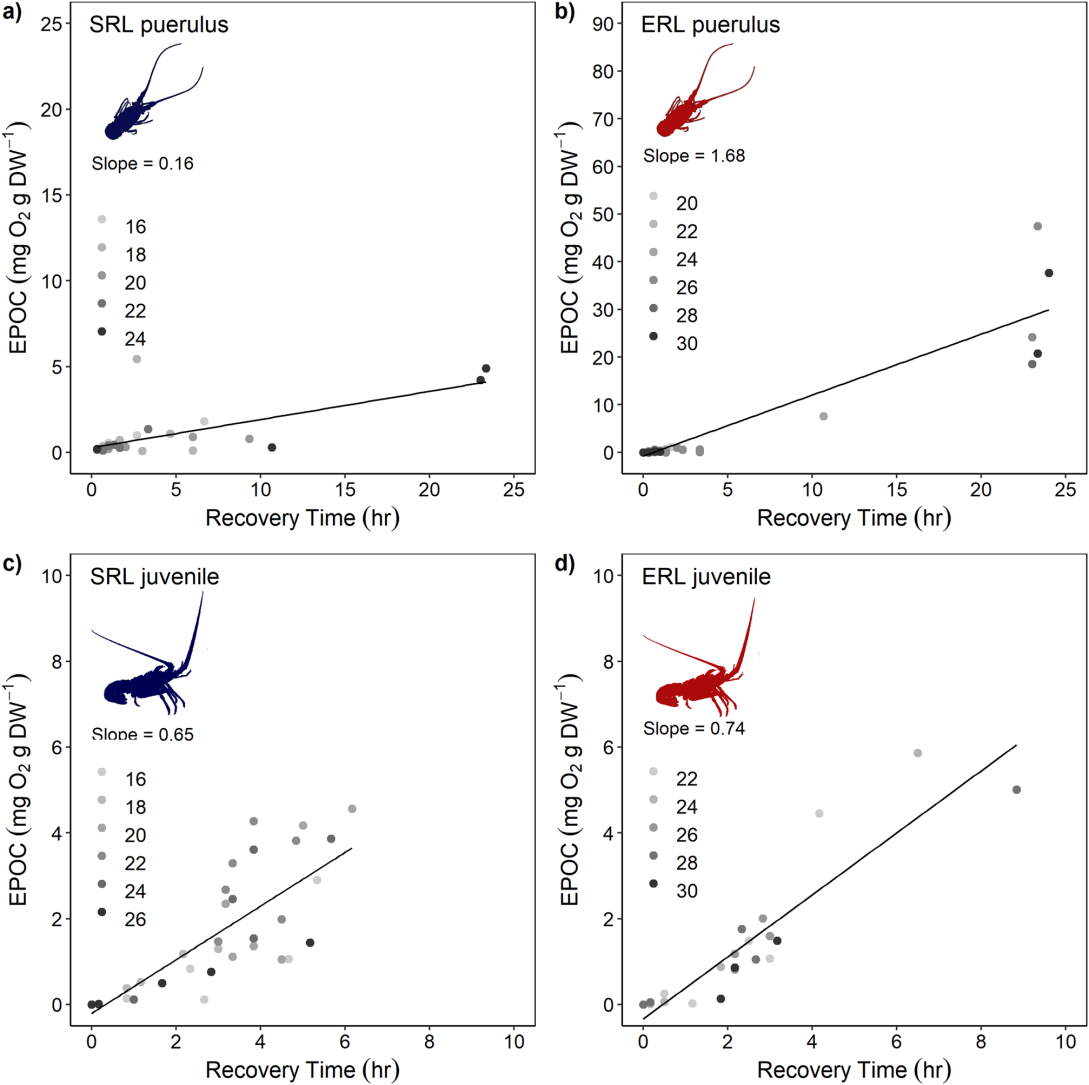
Correlation between recovery time and excess post-exercise oxygen consumption (EPOC) for *Jasus edwardsii* (SRL) and *Sagmariasus verreauxi* (ERL) where (**a**) shows *J. edwardsii* pueruli, (**b**) *S. verreauxi* pueruli, (**c**) *J. edwardsii* juveniles and (**d**) *S. verreauxi* juveniles. Slope value indicates the slope (m) of the regression line y = mx + c. Greyscale points correspond to temperature treatment. Please note the difference in scale for puerulus EPOC values.

Recovery time, the time taken to recover back to oxygen consumption values within two standard deviations of routine metabolic rate, exhibited non-significant relationships with temperature for *J. edwardsii* pueruli and juveniles and *S. verreauxi* juveniles (Supplementary Table S1, Supplementary Fig. S3). *Sagmariasus verreauxi* pueruli were analysed using a binomial response model due to the divide between individuals that recovered quickly and those that did not (Supplementary Fig. S3, Supplementary Table S1). This binomial response model showed a significant effect: with increased temperature, a quick recovery to routine metabolic rate was less likely (Wald test, χ^2^ = 6.7, *p* = 0.010). Excess post-exercise oxygen consumption (EPOC) recovery measurements exhibited non-significant relationships with temperature for *J. edwardsii* pueruli and juveniles and *S. verreauxi* juveniles, again with only *S. verreauxi* pueruli having a significant relationship with temperature, in this case an exponential relationship between recovery time and temperature (Supplementary Fig. S4, Supplementary Table S1). While non-significant, of note is the decline in EPOC in *J. edwardsii* juveniles at the highest temperature treatment. Results of Pearson correlation tests showed significant positive relationships between recovery time and the magnitude of EPOC for both species and life stages (Fig. 6, *J. edwardsii* pueruli, r(29) = 0.668, *p* = 2.48e^−5^; *S. verreauxi* pueruli, r(37) = 0.838, *p* = 2.89e^−11^; *J. edwardsii* juveniles, r(32) = 0.794, p = 2.04e^-8^ and *S. verreauxi* juveniles r(19) = 0.913, *p* = 7.31e^−9^). The slope of the correlation indicates that it takes more oxygen to recover for *S. verreauxi* pueruli than it does for *J. edwardsii* pueruli (1.68 for *S. verreauxi* compared to 0.16 for *J. edwardsii*, Fig. 6). This pattern was also observed in the juveniles, however, the magnitude of difference in the slope is less pronounced. Also note that the trials were stopped at 24 h post-exercise which accounts for the clustering of points around this time. These indicate the individuals that did not recover to within two standard deviations of routine metabolic rate before the trials were completed.

The number of escape responses (measured as the total number of escape responses to stimuli over the period of the chase experiment) exhibited by both species and life stages was non-significant with temperature, except for *J. edwardsii* juveniles that showed a significant linear decline in the number of escape responses as temperatures increased (Supplementary Fig. S5, Supplementary Table S1). *Jasus edwardsii* juveniles also had significantly higher numbers of escape responses than *S. verreauxi* juveniles (Welch two sample t-test; t(52.24) = 4.35, *p* = 6.42e^−5^).

A few mortalities were also recorded during the trials that should be noted. These included *S. verreauxi* pueruli, where one, one and three individuals died at 26, 28 and 30 °C treatments, respectively. These mortalities all occurred after the chase exercise period of the respirometry/escape speed trials, prior to the completion of the final 24 h of the respirometry experiments. There were two mortalities for *S. verreauxi* juveniles, both at 30 °C, and both occurred after the chase period. *Jasus edwardsii* had three mortalities, all at 26 °C, with two pueruli dying before the chase period, and one shortly after.

## Discussion

Our experiments show that thermal pattern, optima and magnitude of response vary between multiple performance measures, life stage and species and highlights that a single measure of performance does not predict whole organism performance^14,19,20,34^. This supports a coordinated assessment of multiple and life-stage specific thermal performance measures to identify performance attributes that are most appropriate to predict the effects of environmental change on species redistribution. Overall, *S. verreauxi* had warmer temperature performance optima for metabolic rates and escape speeds tested for pueruli and juveniles respectively, however *J. edwardsii* had higher magnitudes of response (i.e. faster) for escape speeds. With further ocean warming off Tasmania, it is likely that *S. verreauxi* will continue to expand its range and increase in abundance in areas it was previously scarce.

### Mismatch of thermal optima between performance measure, life stage and species

As hypothesised, *S. verreauxi* displayed warmer optimal temperatures for aerobic scope than *J. edwardsii*, for both pueruli and juveniles (Fig. 3). However, while *S. verreauxi* had warmer optimal temperatures for escape speed, the magnitude of both maximum and average escape speeds were higher in *J. edwardsii* than *S. verreauxi* across all tested temperatures, including those at which the species performances overlap (Figs. 4 and 5). In addition, performance measure optima also varied between life stages within species. Thermal optima were warmer for puerulus than the juvenile stages (Table 1). Thermal response of performance traits differed also within the same species. *Sagmariasus verreauxi* thermal optima were warmer for aerobic scope than escape speed, though for *J. edwardsii*, they were similar (Table 1). The observed variation of thermal optima across performance traits, life stage and species suggests; (1) that temperatures for species optimal performance vary depending on which performance trait is most critical to a particular species, life stage and the predominant ecosystem pressure; (2) that a single measure of performance at a single life stage will not accurately predict whole animal performance over its entire life span, and (3) that thermal history alone may be misleading to predict performance outcomes. Selection of experimental performance traits, therefore, requires careful consideration of the species’ ecological setting and life stage to achieve more accurate estimates of climate driven distribution shifts.

### Aerobic scope

*Sagmariasus verreauxi* had warmer thermal optima for aerobic scope than *J. edwardsii* (Fig. 3). This likely reflects the different thermal histories of both species, with *J. edwardsii* originating from cooler temperate waters and *S. verreauxi* from warm-temperate to subtropical waters. Aerobic scope has been proposed as a way to predict effects of ocean warming on individual species, however, there is evidence for and against its application in this way^16,31–33^. While aerobic scope may not be a unifying principle for performance in all species, it is still a highly valuable measure of metabolic performance and can inform us of potential directions of performance change under ocean warming scenarios. Using this assumption, with continued ocean warming around Tasmania it is likely that *S. verreauxi* will further extend its range into southern Tasmania, as well as increase in abundance, as temperatures become more favourable in terms of their aerobic scope. Further warming and extension of the East Australian Current into Tasmania will likely facilitate both the transport of larvae into the area and warming for over-winter survival in an area previously too cold for puerulus to settle and survive as juveniles^48,49^.

### Escape speed

While *S. verreauxi* have higher aerobic scope magnitudes and warmer thermal optima than *J. edwardsii*, *J. edwardsii* exhibited faster escape speeds across most temperatures including at the crossover temperatures (20–26 °C), which *J. edwardsii* may experience with further warming within its current distribution range (Fig. 4). Faster escapes by *J. edwardsii* indicate significant ecological pressure on this trait during this early life stage. The spiny lobster life cycle is characterised by a long pelagic larval duration before pueruli undertake a costly swim from offshore to near coastal environments to settle into suitable habitats^54^. This settlement process is fraught with danger for pueruli that have to avoid the ‘wall of mouths’ phenomena that faces the larval stages of many different species recruiting into new environments^55,56^. Therefore escaping predator attacks during larval settlement is essential to recruit to and sustain a local population^54^. Because *J. edwardsii* pueruli have the potential to escape faster than *S. verreauxi* they possess a competitive advantage that may influence settlement and recruitment success of the species, based on the assumption that they have the same predators.

### Aerobic versus anaerobic performance traits

The discrepancy of thermal optima between aerobic scope and escape speed may be explained by their vastly different power mode. Aerobic scope is purely supported by aerobic energy provision, taking place in mitochondria that produce ~ 1.5–2.5 adenosine triphosphate (ATP) molecules per consumed oxygen molecule via oxidative phosphorylation^57^. Aerobic scope reflects a complex composition of numerous oxygen dependent processes from the cellular- up to the systemic level^58^. Consequently, as a high-level performance trait, thermal tolerance of aerobic scope results from the cumulative interaction of thermal tolerances of each of these sub-processes, forming an overall thermal tolerance curve which is more constrained than its individual sub-processes. In contrast, escape bursts by decapod crustaceans or fishes, are mainly powered by anaerobic white muscle^40^. Here ATP is derived from ATP stored in the form of phosphoarginine (in crustaceans) as well as by converting glycogen to lactate by anaerobic glycolysis^39,59^. Phosphoarginine powers the first strong burst followed by the weaker burst, which are sustained by glycolysis. This process is strictly time limited until phosphoarginine and glycogen stores are exhausted and muscles become too acidic to operate^39^. Anaerobic white muscle bursts can be considered as a low-level performance trait, as it is largely driven by cellular enzyme reactions and cellular substrate diffusion. Low level cellular processes are less complex and therefore considered to operate at broader temperature scales^58^, and if not constrained by generic body functions, will determine the overall animal response. This would explain why optimal temperature for escape bursts of spiny lobsters divert from those of aerobic scope, as well as why escape bursts (i.e. escape speed and number of escape response, Fig. 4 and Supplementary Fig. S5) show very little response to temperature changes within the measured temperature range.

### Links between opposing performance traits

Operation and thus ecological outcomes of aerobic scope and escape burst are independent from each other. However, escape capacity becomes dependent on aerobic scope, following bursts, during recovery when phosphate and glycogen stores need to be refilled and accumulated lactate and protons to be removed using energy (ATP) supplied from aerobic metabolism (c.f. oxygen debt^60,61^). In this case, aerobic scope defines if and how fast anaerobic energy stores can be recharged, and consequently how frequent animals can face recurring predator attacks.

Our results showed that recovery following escape bursts varies in both time and the amount of oxygen required to pay off the oxygen debt (excess post-exercise oxygen consumption = EPOC) between individuals within temperature treatments. Recovery in juvenile spiny lobsters did not differ between *J. edwardsii* and *S. verreauxi* and showed a gradual distribution of recovery rates among individuals, indicating that juvenile spiny lobsters of both species exhibit similar capacity to recover from escape responses. Similarly, many pueruli of *J. edwardsii* and *S. verreauxi* recovered within similar time frames. However, pueruli exposed to high temperatures included individuals that either recovered quickly (in less than 3 h) or did not recover for the full 24 h of trials, with this result most pronounced in *S. verreauxi* (Supplementary Fig. S3). This suggests that the individuals that recover their oxygen debt more quickly might be less susceptible to predation at higher temperatures than others, driving selection of this particular phenotype^62,63^. Further, *S. verreauxi* required disproportionally more oxygen to recover an oxygen debt at higher temperatures than *J. edwardsii* (Slope of 1.68 compared to 0.16, Fig. 6). This indicates a stronger reliance of *S. verreauxi* pueruli on aerobic scope and sufficient ambient oxygen levels at higher temperatures. These differences in individual responses indicate high intra- and moderate inter-specific variation among spiny lobsters, which implies opportunity for natural selection to favour phenotypes better adapted to warming oceans^62,63^.

### Predicting range shifts and species interactions

For both measures of performance, and both life stages, temperatures can be identified where the thermal performance curves of the two species overlap i.e. for puerulus its approximately 18–20 °C. However, due to the variation between performance measures and life stages, predicting a single temperature where overall animal performance may decline or increase, and hence the outcomes of interactions may change, is challenging i.e. where the cross over for puerulus aerobic scope is approximately 18–20 °C, its approximately 24–26 °C for escape speed. Some form of combined measure may be required, however determining the relative importance of each measure to fitness and survival may also be a challenge. Overall, comparing the thermal performance curves of two or more species may allow us to make general predictions of how species relative performances may change under warming. A caveat here is that thermal performance curves may not reveal the indirect effects of species interactions that are not immediately clear^21^. Despite the challenges, incorporating appropriate physiological measures into models are likely to provide a more complete and robust prediction of future changes for species under future climate scenarios^64^.

## Limitations

Conclusions from this study are limited by the following aspects. First, thermal tolerances presented here are indicative of short term, acute temperature changes due to the short acclimation periods, especially for the puerulus stages. This short period may have reduced any chance for phenotypic plasticity to occur. While longer acclimation periods may have allowed for phenotypic plasticity to occur (i.e. potentially apparent as reduced metabolic rates following warm-acclimation)^63,65^, chronic exposure to elevated temperatures may also have resulted in negative effects on physiology, such as decreased fitness, decreased body condition or decreased aerobic scope. Therefore, these results do not necessarily reflect changes in performance over longer time periods where species plasticity and acclimation may mitigate the negative effects of a warming environment^66^. This said, these results are more relevant to shallow tidal waters, movements across steep thermoclines or shorter-term warming events such as marine heatwaves. In recent decades, marine heatwaves have increased in number and severity in south-east Australia, including a particularly strong event over the Austral summer of 2015/16^67,68^. This trend is likely to continue in the future^68^ and the results from the short experiment presented here might be indicative of changes observed during a heatwave event. Second, thermal performance curves do not incorporate other aspects such as mortality rates during the derivation of the curves. For example, *S. verreauxi* pueruli had the warmest thermal tolerances of the study at ~ 27 °C for aerobic scope but this trial also observed the highest mortality rate of five individuals. Long term exposure to such higher temperatures may further increase mortality and decrease thermal optima. Thus, caution should be used when interpreting thermal tolerance results as not all measures are representative of the overall performance of a whole group of individuals. Finally, this study is constrained by use of *S. verreauxi* individuals bred at the IMAS aquaculture facility while *J. edwardsii* were wild caught. This may introduce bias in that *S. verreauxi* individuals were not subject to predation pressure during their earlier life stages. This may also indicate that performance for this species may be underestimated as the tested individuals have not had to survive predation through their larval and settlement stages, thereby not filtering out the most successful individuals as would be the case for *J. edwardsii*. However, as intra-specific variation was still high, we can assume that we have captured an accurate representation of effects of temperature on measures of performance in *S. verreauxi*.

## Conclusion

Thermal optima vary between performance measure, life stage and species. Our data emphasize that it is critical to evaluate multiple performance measures, relevant to the specific ecosystem pressure during developmental ontogeny, to better predict the effects of environmental change on species fitness, survival, and distribution. Developing a metric to take multiple measures into account with relevant importance would be valuable, however developing this overall measure would be challenging.

We found that *S. verreauxi* have warmer aerobic scope thermal optima than *J. edwardsii*, indicating that with continued warming in south east Australia, particularly in Tasmania, *S. verreauxi* have the thermal capacity to expand their range and increase in abundance. However, *J. edwardsii* had faster escape speeds and can potentially avoid predation better than *S. verreauxi* at common crossover temperatures. The relative impact of aerobic scope and predator escape capacity will vary with life stage (e.g. larvae, adult) and specific ecosystem pressure (e.g. predator abundance). The current average summer temperatures off Tasmania’s east coast are approximately 18 °C^69^. With a projected ocean temperature increase of 3.5 °C by 2100^70^, it would put *J. edwardsii* close to a temperature at which their aerobic scope and escape speed performance may begin to decline. While we can predict potential individual changes in species performance based on abiotic factors, what these changes may mean for any competitive interaction between the two species is yet to be determined in any newly shared environment.

While thermal performance curves are useful in illustrating how a single species may react to warming, they may not reveal the potential indirect effects of changing species interactions that might occur, such as shifts in competitive success over food or shelter. These indirect effects can be illustrated by experimental manipulations^21^. In addition, incorporating thermal performance data into modelling approaches may be able to elicit some of these indirect effects that are not clearly seen or predicted using individual species thermal performance curves^71^. Future studies should directly quantify the outcomes of a direct interaction between the local and range-shifting species to determine if measures of individual thermal tolerances can predict changes in species interactions under future ocean warming scenarios.

## Methods

### Animal collection and holding

*Sagmariasus verreauxi* pueruli were cultured from eggs at the Institute for Marine and Antarctic Studies (IMAS)^72,73^ and held prior to experimentation as per Twiname, et al.^19^. *Sagmariasus verreauxi* individuals were raised from eggs through to puerulus stage larvae in a mixed pool of recruits at temperatures between 21 and 23 °C. Upon metamorphosis to pueruli, individuals were haphazardly selected for this experiment and placed in individual cylindrical vessels (300 ml) suspended in a 68 L polypropylene sump supplied with temperature-controlled, filtered, ozonated seawater. *Sagmariasus verreauxi* pueruli were held at their rearing temperature for seven days prior to any experimentation. This was to ensure reared *S. verreauxi* were of a similar age to wild-caught *J. edwardsii*. *Jasus edwardsii* pueruli (*n* = 29) were collected monthly from puerulus collectors located at Bicheno (41.85° S, 148.26° E), Iron Pot (43.06° S, 147.42° E) and Recherche Bay (43.55° S, 146.90° E), along the eastern and southern coasts of Tasmania, Australia. Ambient temperatures at collection ranged from 12–18 °C depending on time of collection between July 2016–March 2017. Pueruli collected were transported back to the IMAS and placed into vessels with the sample water conditions (temperature controlled, filtered, ozonated seawater) as per *S. verreauxi* pueruli. They were held at the sampling temperature (i.e. ambient SST at time of collection) for one day prior to experimentation. Juvenile *S. verreauxi* (*n* = 24, 42.7–59.5 mm carapace length) were also cultured from eggs at IMAS from the previous year, and *J. edwardsii* juveniles (*n* = 36, 37.2–50.8 mm carapace length) were selected from a stock of individuals collected in previous years as pueruli and reared at the IMAS facilities. Juvenile lobsters of each species were held separately in 5000 L tanks supplied with unfiltered sea water at ambient temperatures. Juvenile lobsters were fed 2–3 times per week to excess with fresh or thawed blue mussels (*Mytilus galloprovincialis*). Individuals of both species were selected for trials based on size and time since moulting. As *S. verreauxi* grow faster than *J. edwardsii*, individuals selected for the trials were of similar size but were different ages (i.e. 1-year old *S. verreauxi* compared to 2-year-old *J. edwardsii*). Once a lobster of either species moulted, they were transferred into 65 L holding tanks, species separated, at ambient temperatures for one week prior to experiments to reduce effects of moulting on metabolic or behavioural results. The holding tanks were supplied with temperature controlled, filtered, ozonated seawater and the juvenile lobsters were fed daily to excess with fresh or thawed blue mussels. These experimental trials were conducted on invertebrate crustaceans and required no animal ethics, however utmost care was given to the animals. *Jasus edwardsii* pueruli were collected under the DPIPWE permit numbers 15108 and 16104.

### Experimental procedure

Trial temperatures were achieved by increasing or decreasing the water temperature, via a submersible 2000 W heater (custom made by Istra Elements, Australia), by 2 °C per 24 h until the trial temperature was achieved. The 2 °C heating or cooling per 24 h was within the daily temperature variance experienced by lobsters in natural environments of 0.3–4.6 °C^69^. Pueruli had a two-day acclimation period and juveniles a minimum of seven days acclimation to trial temperatures before any experiments commenced. Pueruli had a shorter acclimation period due to the shorter larval stage duration. For *S. verreauxi* and *J. edwardsii* pueruli and juveniles, temperature effects on metabolism and escape response were investigated over different thermal ranges at 2 °C intervals. Sample sizes for *S. verreauxi* pueruli were 6, 6, 6, 9, 10 and 8 individuals tested at 20, 22, 24, 26, 28 and 30 °C, respectively. For *S. verreauxi* juveniles, sample sizes were 5, 5, 4, 5 and 3 for 22, 24, 26, 28 and 30 °C, respectively. Due to constraints on the number of available suitably sized *S. verreauxi* juveniles, only five temperatures were investigated (22–30 °C by 2 °C intervals). The low sample size of three at 30 °C was due to two juvenile mortalities at this trial temperature, occurring after the chase period. These were excluded from the statistical analyses. Sample sizes for *J. edwardsii* pueruli were 6, 7, 7, 6, 4 and 0 tested at 16, 18, 20, 22, 24 and 26 °C, respectively. Due to the short puerulus intermoult period, only three *J. edwardsii* pueruli replicates were able to be conducted at 26 °C i.e. only three individuals did not moult into juveniles during the acclimation period. However, all of these three replicates died during the respirometry trials, two before the chase period, and one afterwards. For *J. edwardsii* juveniles, the samples size was six for each temperature treatment of 16, 18, 20, 22, 24 and 26 °C. For each of these temperatures, both aerobic metabolism and escape speed trials were conducted concurrently. Lobsters were introduced into their respirometry chambers and left for 16 h. From this phase, routine and standard metabolic rates were measured. At approximately 16 h, lobsters were removed, one at a time, and placed into a chase area. The lobsters were chased, and escape responses of escape speed and number of escapes were measured during this phase. After this chase period, lobsters were placed back into their respective chamber and left for a further 24 h, from which active metabolic rate, recovery time and excess post-exercise oxygen consumption (EPOC) values were measured.

### Respirometry

Intermittent flow respirometry was used to investigate thermal effects on the lobster oxygen consumption rates (*Ṁ*O_2_) as a measure of aerobic metabolism. For the pueruli, intermittent flow respirometry protocols were similar to that described as by Fitzgibbon, et al.^51^ (see Supplementary Fig. S1 for the respirometry set up). As the puerulus stage is a non-feeding larval stage^54^, fasting to reduce the effects of specific dynamic action was not required. Pueruli were placed in 19 ml glass respirometry chambers (Loligo horizontal mini chambers, Denmark) submerged in a 3.5 L sump supplied with ozonated, temperature-controlled seawater. Peristaltic pumps (Harvard Apparatus Mini-Peristaltic Pump II, USA) provided continuous recirculation past the oxygen sensors as well as chamber flushing every 10 min. Dissolved oxygen measurements were taken every 10 s using a fibre optic dissolved oxygen analyser (PreSens OXY-4 Mini multichannel fibre optic oxygen transmitter, Germany), and remained between 75 and 100% saturation for the duration of the trials. Puerulus trials were started at approximately 16:00 and ran overnight for a period of 16 h. During this period, minus the first two hours of data to let the pueruli settle into the chambers, standard and routine metabolic rates were measured. At approximately 08:00 the following day, pueruli were removed individually and placed into a 100 L, 59 cm diameter chase arena (see Supplementary Fig. S2). The pueruli were manually chased for a period of approximately nine minutes (timed to coincide with the 10-min open: close cycle) before being placed back into their respective chambers for another 24 h, where active metabolic rates and recovery parameters were measured.

For the juveniles, a similar protocol was followed but used a larger system similar to that described by Jensen et al.^74^ (see Supplementary Fig. S1 for the respirometry set up). Prior to respirometry trials, juveniles were fasted for 48 h to reduce the effects of specific dynamic action on metabolic rates. A 500 L sump was supplied with temperature controlled, filtered, and ozonated seawater and fitted with an additional submersible heater to maintain water temperature, as well as two air stones for water circulation and aeration. Four custom-made Perspex respirometry chambers were placed in the sump, each connected to two pumps, a flush pump and a recirculation pump, ensuring water was pumped continuously across a dissolved oxygen probe (Hach Intellical LDO101 Luminescent Dissolved Oxygen sensor, USA), as part of the tubing exiting the chamber. Dissolved oxygen measurements were taken every 30 s during a 5 min open: 5 min closed cycle. Flush cycles and chamber sizes were designed to ensure that the dissolved oxygen levels never dropped below 75% saturation. A larger sized chamber (1000 ml) was used for individuals > 50 g wet weight and a smaller chamber (485 ml) for lobsters < 50 g. Pilot trials confirmed that there was no significant difference in routine metabolic rate measurements taken between the two different sized chambers (paired t-test, t(91) = 1.66, *p* = 0.179). As per the puerulus trials, juvenile experimental trials were started at approximately 16:00 and ran overnight for a period of 16 h, from which standard and routine metabolic rates were measured. At approximately 08:00 the following day, juveniles were removed individually and placed into a 300 L, 150 cm diameter chase arena. The juvenile lobsters were manually chased for a period of approximately 13 min (timed to coincide with the five-minute open: close cycle) before being placed back into their respective chambers for another 24 h, where active metabolic rates and recovery parameters were measured.

Metabolic rates were determined by protocols similar to those described by previous studies on spiny lobsters^19,20,51,74,75^ and by Clark, et al.^31^. Routine metabolic rate was calculated as the mean of *Ṁ*O_2_ measurements during the first 16 h of the trial, with the first two hours of experimental data excluded to account for chamber acclimation. Standard metabolic rate was calculated as the mean of the lowest 10% *Ṁ*O_2_ measurements during the trial, and active metabolic rate was the mean of the two highest *Ṁ*O_2_ measurements during the post-exercise period^31^. Time to recovery was taken as when the *Ṁ*O_2_ came back to within two standard deviations of routine metabolic rate after the exercise period. Excess post-exercise oxygen consumption was calculated using the area under the *Ṁ*O_2_ curve after exercise to when it returned to routine metabolic rate + two standard deviations.

### Escape speed trials

The escape speed trials were filmed when the individuals were removed from their chamber and placed in a chase arena supplied with temperature-controlled, filtered, and ozonated seawater at the same temperature as the respirometry trial from the same sump. The escape responses were filmed using stereo video cameras (GoPro HERO4, USA), mounted on a custom-made stainless-steel frame holding the cameras at 15 degrees inwards from the perpendicular to the bar, allowing for overlap of the two camera fields of view (see Supplementary Fig. S2). The escape response videos were filmed at 240 frames per second and 720p (1280 × 720 pixels) resolution. The high frame rate was used to ensure escape speed was accurately recorded and the stereo system to ensure accuracy when the lobsters did not escape in the direction of the camera field of view^76^. The lobsters were chased for a period of time before being placed into their original chambers to continue the post-exercise metabolic trials. Chasing involved gently tugging on the antennae of the lobsters or gently prodding the legs and bodies by hand. Escape responses were recorded when stimulation caused a tail-flick response. When the lobster ended the flicking response, the escape was deemed over. These responses were then then counted to give a total number of escape responses. The maximum escape speed was the single fastest response calculated per individual. The average escape speed was the mean of all escape responses recorded for each individual. For the pueruli, the chase arena consisted of the camera system mounted above a 100 L, 59 cm diameter tank. The chase period was approximately nine minutes, timed to coincide with the 10-min open: close cycle. Once this was complete, the pueruli were placed back in their respective chambers to complete the respirometry trials. For the juvenile escape speed trials, the camera system was mounted above a 300 L, 150 cm diameter arena. At 08:00, juvenile lobsters were individually removed from their chambers, placed in the arena and manually chased for a period of approximately 13 min. This timing allowed for the lobsters to be chased and replaced back into their respective chamber before the chambers began a new closed cycle at 15 min. Chase times for both life stages were chosen to coincide with the respirometry flush cycle, allowing lobsters sufficient time to become exhausted, and immediate oxygen consumption measurements to be taken once the lobster was placed back into its chamber^19,20,51,75^. An escape response was recorded when the lobster initiated a tail flick response to stimuli and included all tail flicks until the lobster came back to a stop.

### Data and statistical analysis

Data and statistical analyses were performed using MS Excel, EventMeasure Stereo (SeaGIS software, Australia, www.seagis.com.au) and the R version 3.5.1^77^.

Oxygen consumption rates were calculated in Excel and analysed using regression models in R. Oxygen consumption rates were calculated using whole animal dry weight (DW), estimated as 22% of the wet weight measured using an analytical balance^75^. Background respiration was measured simultaneously in an empty chamber during all trials and was accounted for by subtracting background oxygen consumption values from the oxygen consumption values of the trial measurements prior to correction for mass. Final corrected metabolic rates are expressed as mg O_2_ gDW^−1^ h^−1^. Recovery measures were calculated as the time and magnitude taken for metabolic rates to come back within two standard deviations of routine metabolic rate. Magnitude of EPOC was the sum of the area under the curve between measured metabolic rates after exercise minus the routine metabolic rate plus two standard deviations.

The stereo video footage was analysed using EventMeasure Stereo with distance travelled calculated in three dimensions (X, Y, Z coordinates) while the frame rate gave the time taken to complete the escape response. Body velocity or escape speed (in m s^-1^), was then calculated using the adapted Pythagoras’s theorem equation below (1):

1 $$ Velocity = \frac{{\sqrt {\left( {X_{end} - X_{start} } \right)^{2} + \left( {Y_{end} - Y_{start} } \right)^{2} + \left( {Z_{end} - Z_{start} } \right)^{2} } }}{Number\;of\;video\;frames} $$

where X, Y and Z are the three-dimension coordinates and are separated into ‘start’ position and ‘end’ position of the escape response, and the number of video frames taken to complete the responses from start to end frame.

Regression modelling was used to analyse relationships between measured variables and temperature. Metabolic rates, EPOC and escape responses (maximum and average speeds as well as number of escape responses) were assessed for normality using residual plots and Shapiro–Wilk’s normality tests, assessed for homogeneity of variance using Levene’s test, and analysed using the appropriate linear, generalised linear, and linear mixed effects models^78^. For the mixed effects models, respirometry chamber was included as an error term to account for any effect of the chamber on respirometry measures. Different regression models were tested (i.e. linear, quadratic, exponential etc.) and the best model fit was determined using Akaike’s Information Criterion (AIC). Recovery time for *S. verreauxi* pueruli was analysed using a binomial response model due to its unique response. Where appropriate, optimal temperatures (T_opt_) for the thermal performance curves were calculated using the first derivative of the quadratic equation from the regression analysis. Pearson’s correlation tests were used to identify response parameters that were significantly correlated. All figures were produced using ggplot2^79^.

## Supplementary information

Supplementary information.
