# Supplementary material for: Mismatch of thermal optima between performance measures, life stages and species of spiny lobster
Source: Sci Rep. 2020 Dec 4;10:21235. doi: 10.1038/s41598-020-78052-4 (PMC7718242; doi:10.1038/s41598-020-78052-4)
Supplement: Supplementary file 1 — Supplementary information. [file 41598_2020_78052_MOESM1_ESM.pdf]

Supplementary Information

Mismatch of thermal optima between performance measures, life stages and species of spiny lobster.

Samantha Twiname[1]*, Quinn P. Fitzgibbon[1], Alistair J. Hobday[2], Chris G. Carter[1], Michael Oellermann[1], Gretta T. Pecl[1]

Supplementary Table S1. Regression analyses of performance responses to temperature of *Sagmariasus verreauxi* (ERL) and *Jasus edwardsii* (SRL). Analyses in the form of linear (y = ax + b), exponential (y = exp(ax + b)), quadratic ($y = ax^2 + bx + c$) and polynomial ($y = ax^3 + bx^2 + cx + d$) regressions. * indicates significance at $p < 0.05$.

	Regression	a	b	c	d	R^2	F-statistic	df	p
**Standard metabolic rate**									
ERL puerulus	Linear	0.075	-1.010	-	-	0.586	62.360	44	0.000*
ERL juveniles	Polynomial	0.003	-0.199	5.063	-42.600	0.523	6.576	18	0.003*
SRL puerulus	Linear	0.048	-0.255	-	-	0.374	18.33	28	0.000*
SRL juveniles	Polynomial	0.001	-0.059	1.180	-7.557	0.659	20.600	32	0.000*
**Routine metabolic rate**									
ERL puerulus	Exponential	0.073	-1.700	-	-	0.633	75.720	44	0.000*
ERL juveniles	Polynomial	0.003	-0.207	5.390	-46.100	0.541	7.062	18	0.002*
SRL puerulus	Linear	0.054	-0.233	-	-	0.451	24.830	28	0.000*
SRL juveniles	Polynomial	0.001	-0.074	1.477	-9.459	0.674	22.060	32	0.000*
**Active metabolic rate**									
ERL puerulus	Exponential	0.064	-0.952	-	-	0.485	41.350	44	0.000*
ERL juveniles	Linear	0.001	0.702	-	-	0.002	0.049	20	0.828
SRL puerulus	Exponential	0.037	-0.475	-	-	0.334	15.540	28	0.000*
SRL juveniles	Quadratic	-0.002	0.088	-0.319	-	0.258	5.746	33	0.007*
**Aerobic scope**									
ERL puerulus	Quadratic	-0.010	0.531	-5.983	-	0.148	3.738	43	0.032*
ERL juveniles	Quadratic	-0.004	0.209	-2.032	-	0.300	4.066	19	0.034*
SRL puerulus	Linear	0.001	0.584	-	-	-0.035	0.027	28	0.871
SRL juveniles	Quadratic	-0.005	0.190	-1.451	-	0.514	17.420	33	0.000*
**Time to recovery**									
ERL puerulus	Exponential	0.184	-4.407	-	-	0.1638	7.248	37	0.011*
ERL juveniles	Exponential	0.069	-1.323	-	-	0.029	0.571	19	0.459
SRL puerulus	Exponential	0.089	-1.100	-	-	0.006	1.181	28	0.287
SRL juveniles	Exponential	0.007	0.637	-	-	0.000	0.016	32	0.901
**Excess post-exercise oxygen consumption**									
ERL puerulus	Exponential	0.308	-8.280	-	-	0.1779	6.491	30	0.016*
ERL juveniles	Exponential	0.102	-2.922	-	-	0.034	0.605	17	0.447
SRL puerulus	Exponential	0.025	-1.253	-	-	-0.032	0.104	28	0.750
SRL juveniles	Exponential	0.130	-2.538	-	-	0.096	3.067	29	0.090
**Maximum escape speed**									
ERL puerulus	Quadratic	-0.005	0.214	-1.720	-	0.131	3.154	42	0.053
ERL juveniles	Quadratic	-0.007	0.331	-2.434	-	0.238	2.960	19	0.076
SRL puerulus	Quadratic	-0.007	0.269	-1.732	-	0.081	2.283	27	0.121
SRL juveniles	Quadratic	-0.005	0.235	-0.968	-	0.097	1.776	33	0.185
**Average escape speed**									
ERL puerulus	Quadratic	-0.002	0.118	-0.926	-	0.131	3.158	42	0.053
ERL juveniles	Quadratic	-0.003	0.120	-0.328	-	0.105	1.118	19	0.348
SRL puerulus	Quadratic	-0.004	0.180	-1.221	-	0.233	5.408	27	0.011*
SRL juveniles	Quadratic	-0.003	0.158	-0.804	-	0.393	10.670	33	0.000*
**Number of escape responses**									
ERL puerulus	Quadratic	-0.149	7.411	-80.008	-	0.053	1.206	43	0.310
ERL juveniles	Quadratic	-0.250	12.460	-141.113	-	0.108	1.154	19	0.337
SRL puerulus	Exponential	-0.011	2.359	-	-	-0.033	0.067	28	0.797
SRL juveniles	Linear	-1.314	47.989	-	-	0.349	18.230	34	0.000*

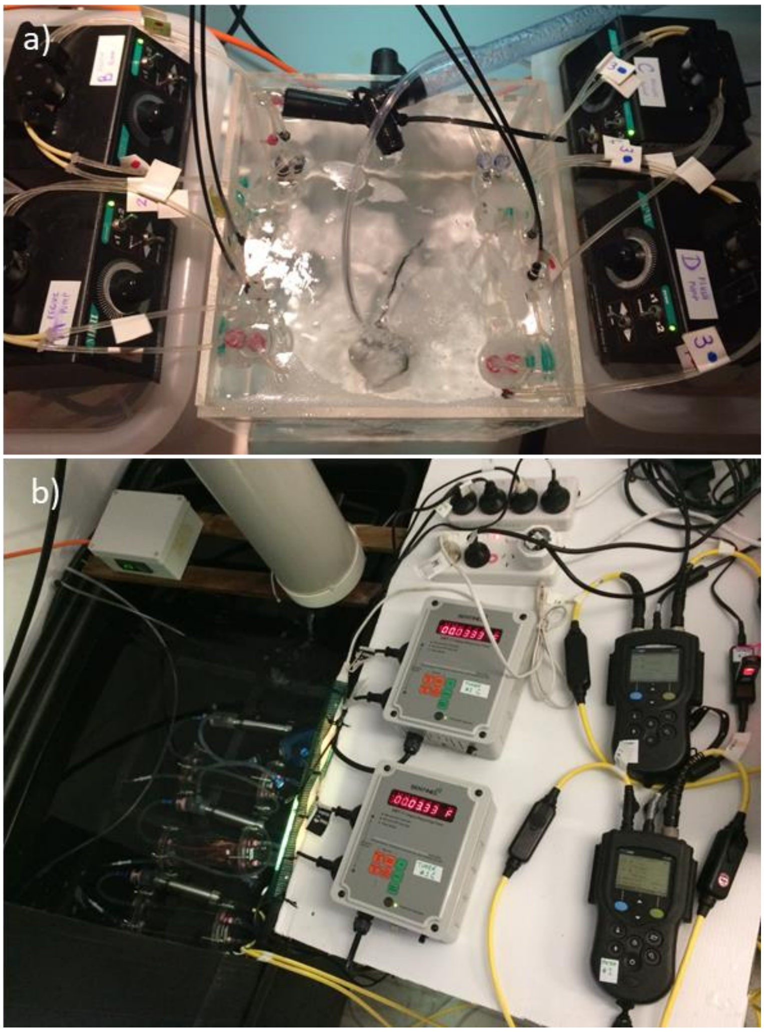

Supplementary Figure S1. The respirometry set up used for a) pueruli, and b) juveniles. Each chamber for both systems was connected to a flush pump, a recirculation pump and oxygen sensor. Image credit S. Twiname.

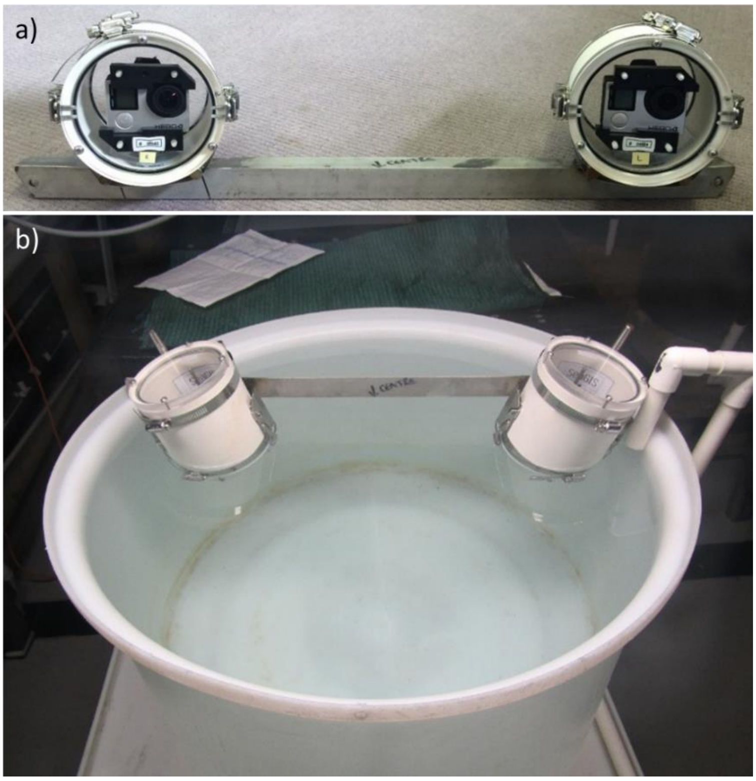

Supplementary Figure S2. Stereo-camera set up used to film puerulus lobster escape responses. Two GoPro cameras fitted in waterproof housings on a stainless-steel bar (a) mounted above a well-lit chase arena (b). A larger system of a similar design was used for recording juvenile escape responses. Image credit S. Twiname.

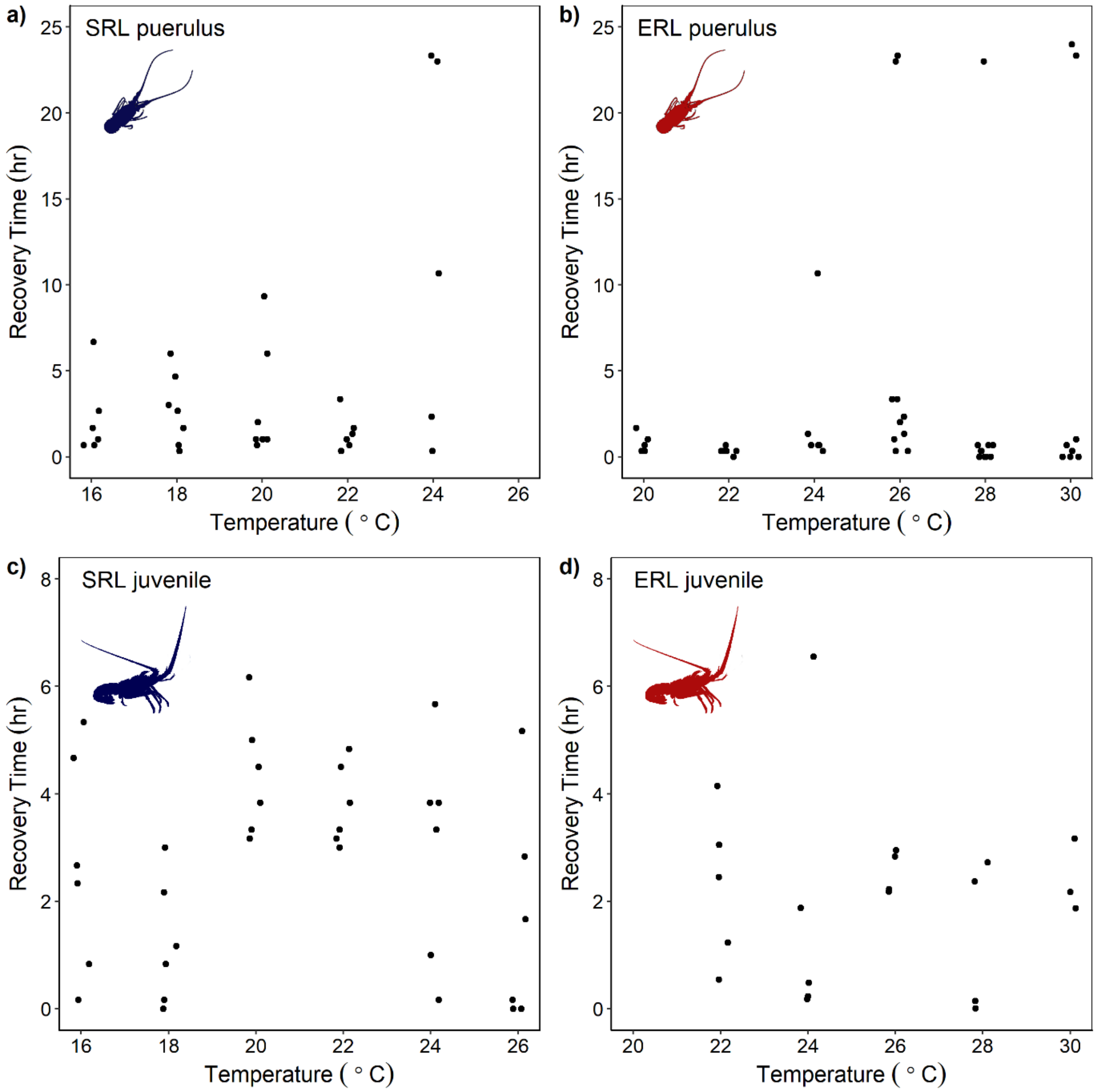

Supplementary Figure S3. Recovery time calculations for *Jasus edwardsii* (SRL) and *Sagmariasus verreauxi* (ERL). Recovery is calculated as the return time to two standard deviations of resting metabolic rate after exercise, where a) shows *J. edwardsii* pueruli, b) *S. verreauxi* pueruli, c) *J. edwardsii* juveniles and d) *S. verreauxi* juveniles. Values are mean ± 1 SE. Sample size ranged from 3 to 10 individuals per temperature treatment. Details for regressions are provided in Supplementary Table S1.

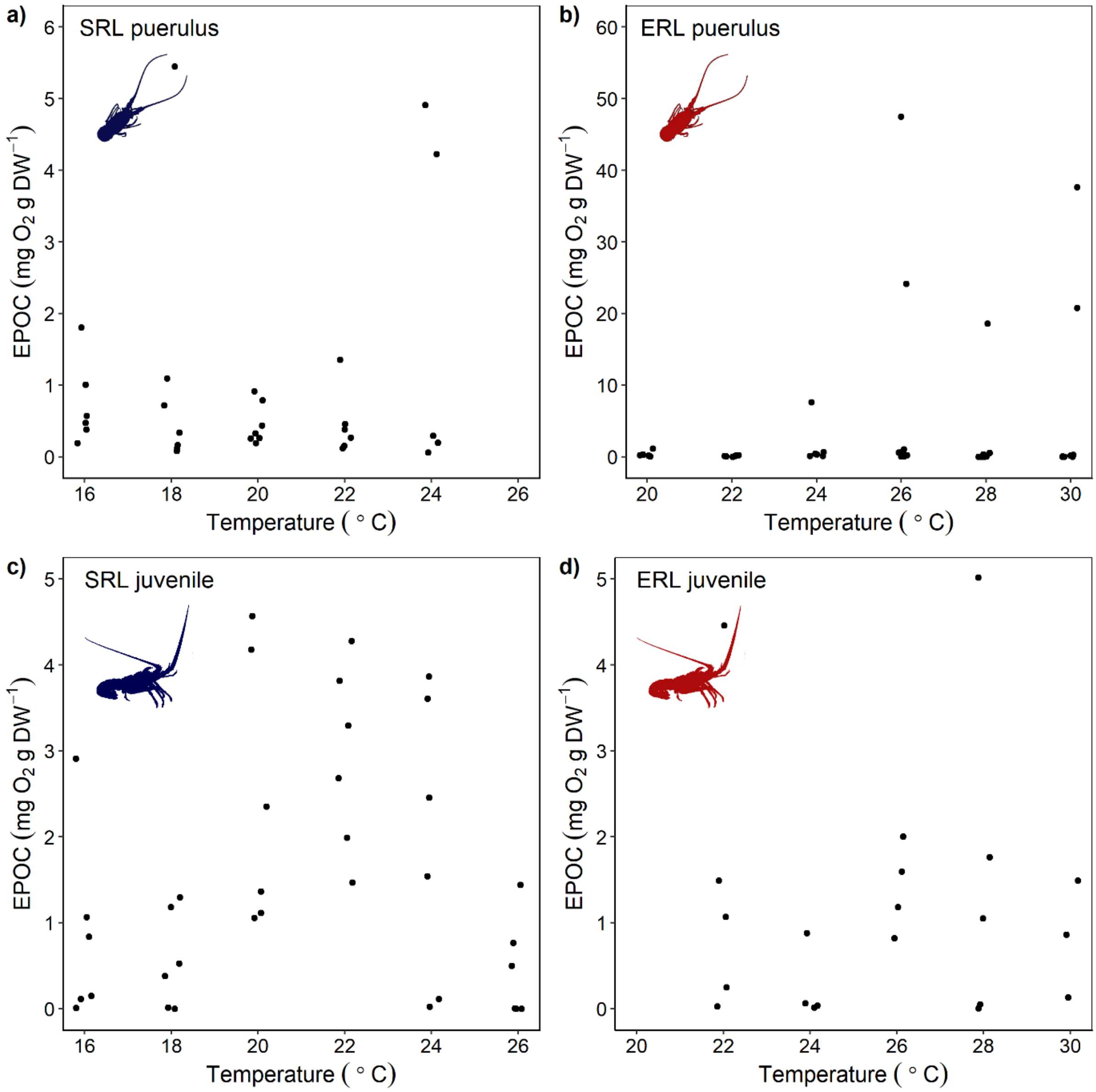

Supplementary Figure S4. Excess post-exercise oxygen consumption (EPOC) for *Jasus edwardsii* (SRL) and *Sagmariasus verreauxi* (ERL). Grid plots are of a) *J. edwardsii* puerulus, b) *S. verreauxi* puerulus, c) *J. edwardsii* juveniles and d) *S. verreauxi* juveniles. Values are mean ± 1 SE. Sample size ranged from 3 to 10 individuals per temperature treatment. Details for regressions are provided in Supplementary Table S1. Please note the difference in scale for puerulus EPOC values.

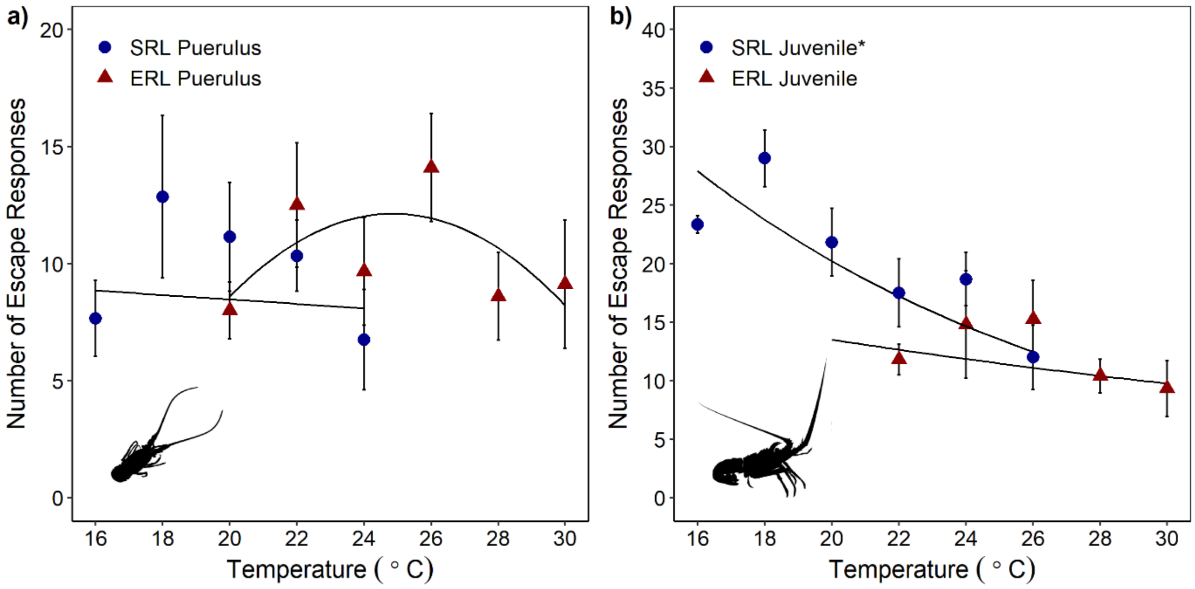

Supplementary Figure S5. Number of escape responses were measured as the number of tail flick responses to stimuli for *Jasus edwardsii* (SRL) and *Sagmariasus verreauxi* (ERL) where a) shows the number of puerulus escape responses and b) the number of juvenile escape responses. Values are mean ± 1 SE. Sample size ranged from 3 to 10 individuals per temperature treatment. Significance at $\alpha = 0.05$ signified by * in legends. Details for regressions are provided in Supplementary Table S1.
